# Principles of management of hand fractures

**DOI:** 10.1177/17504589221119739

**Published:** 2022-11-19

**Authors:** Neophytos Christodoulou, Dimitrios Asimakopoulos, Konstantinos Kapetanos, Matthew Seah, Wasim Khan

**Affiliations:** 1 School of Clinical Medicine, University of Cambridge, Cambridge, UK; 2 Division of Trauma and Orthopaedic Surgery, University of Cambridge, Cambridge, UK

**Keywords:** Metacarpal fractures, Phalangeal fractures, Pain management, Splint, Fracture fixation, Hand therapy

## Abstract

The optimal management of hand fractures requires a multidisciplinary approach. Initial assessment should include a thorough medical history and clinical examination, followed by appropriate radiological imaging. These are crucial in determining the appropriate management. Following joint stabilisation to allow fractures to unite, early mobilisation is needed to maximise the functional restoration of the hand. In this review, the principles of operative and non-operative management of these injuries are discussed.

## Introduction

Hand fractures are breaks in any of the five metacarpals and the associated phalanges of the hand (BSSH 2022). This type of fracture is very common and accounts for, on average, 24,401 cases per year in the UK, according to the Hospital Episodes Statistics database for the period between 2004–2005 and 2013–2014 (Jennison & Brinsden 2019). According to the Health and Safety Executive, with regard to nonfatal injuries to employees in Great Britain, of 481,528 injuries between 2014 and 2021, 17.5% were finger and hand injuries (HSE 2020). Thus, correct management of hand fractures is important to ensure the function is returned. Each finger has three phalanges (the proximal, middle and distal), except for the thumb which has only two phalanges (Moore et al 2015). Although the majority of fractures are closed, approximately 5% are open with a break in the skin at the fracture site (Ng et al 2014). The treatment of most closed fractures is non-operative, by contrast unstable and open fractures generally require surgery.

The multidisciplinary team consists of the preoperative team, the intraoperative team and the postoperative team. The preoperative team, which consists of the nurse practitioner and the A&E physician and surgeon or surgeons (orthopaedic and/or plastic surgeon), are responsible for first assessing the patient in the Accident & Emergency department. The intraoperative team consists of the surgeon, the anaesthetist and theatre practitioners. Finally, the postoperative team, consisting of the ward staff and hand therapist, is in charge of the patient’s rehabilitation. The coordinated input from the aforementioned individuals is essential for the optimal restoration of hand function.

This narrative review outlines the principles of management of adult hand fractures based on the latest guidelines by the National Institute for Health and Care Excellence (NICE) and the British Society for Surgery of the Hand (BSSH). This review considers the perioperative management principles that help restore optimal hand function for a patient with a hand fracture.

## Preoperative management of hand fractures

### Initial management in accident and emergency

#### Patient presentation

This article will focus on the management of both closed and open fractures. Patients with hand fractures usually present to the A&E department following a fall, a sporting incident or a crush injury to the hand (BSSH 2022). On presentation, a thorough history should include details such as the mechanism and time of injury, the degree and duration of any crushing force, if applicable, and whether there is a break in the overlying skin or a penetrating injury. Understanding the patient’s medical history and drug history is important as the presence of comorbidities (eg: diabetes) or tobacco use may impact outcomes. Other key components of the patient history include their hand dominance, occupation and any hobbies which may require dexterity.

#### Clinical examination

**Figure 1:**
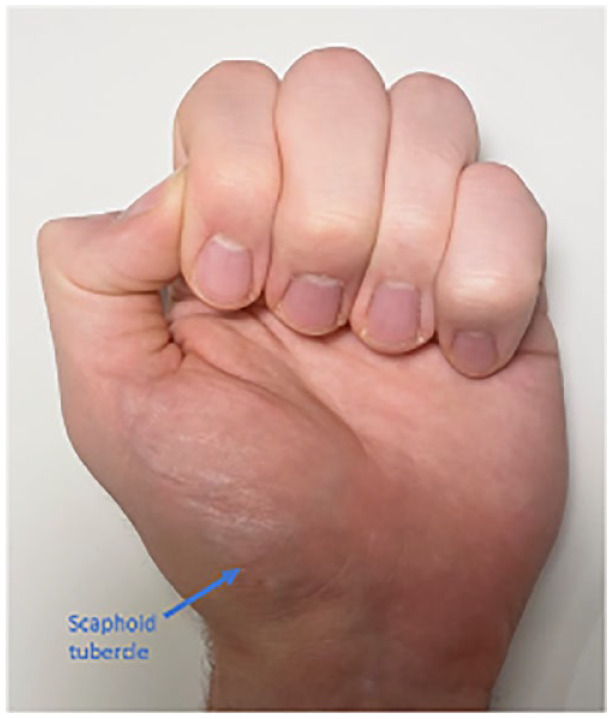
The anatomical position of the scaphoid tubercle, as illustrated by orientation of the digits of a normal hand

Hand examination is facilitated by comparing the injured hand to the uninjured hand, based on Apley’s principles of ‘look, feel, move’ (Solomon et al 2010). Any skin and soft-tissue changes including bruising, redness, wounds, asymmetry or deformity should be noted. Any open wound is inspected to assess the degree of contamination and any potential tendon, ligament or neurovascular injury. Documentation of examination findings is essential and clinical pictures can aid the MDT in their clinical decision-making. In association with the fracture, there may be shortening, angular or rotational deformities. Rotational malalignment is assessed by asking the patient to make a fist and examining the finger flexion cascade, as illustrated in Figure 1. The fingernail should be parallel to the palm and the nails of the other digits. Malrotation should be excluded because it can impair hand function (Green et al 2016). A comparison with the uninjured hand is important, and any previous injuries of the ipsilateral hand which may affect the flexion cascade should be taken into account.

**Figure 2:**
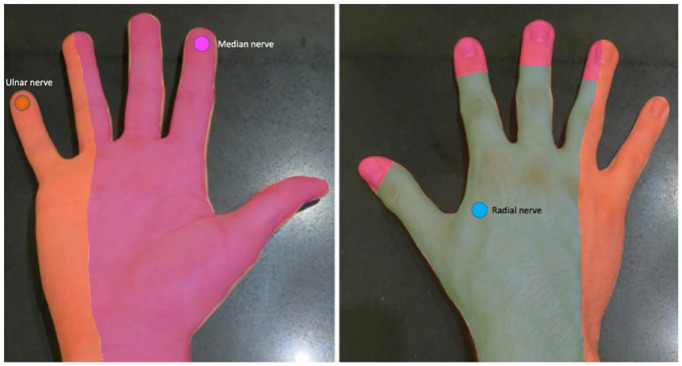
Examination of the sensory nerve supply of the hand, based on Thai et al (2015) The dots represent the discrete testing points for each nerve

Neurovascular assessment must be performed. Extremities should be warm and well perfused, with a normal capillary refill time of two or less seconds, and skin temperature compared with the unaffected side (Odak & Bhalaik 2014). The integrity of the motor and sensory function of the radial, median and ulnar nerves should also be assessed (Thai et al 2015), as shown in Figure 2.

#### Preoperative pain management

Pain assessment should be carried out at the first presentation. This can be carried out in several ways, ranging from published tools like the visual analogue scale (VAS) which may be more useful in children, or by simply asking the patient to rate their pain on a scale of 0 to 10, with 0 being no pain, and 10 the worst possible pain (Delgado et al 2018). Frequent assessment should be carried out depending on pain judgement. Initial pharmacological pain management involves the prescription of non-opioid oral medications for less severe pain and intravenous morphine for moderate-to-severe pain (NICE 2017). Any patient with impaired breathing or cognition will require careful medicine management. Opiates should be avoided in a patient with concurrent head injury (NICE 2021).

#### Imaging modalities

X-rays are the first diagnostic tool of choice to diagnose hand or finger fractures (BSSH 2022). Anteroposterior, lateral and oblique views of the affected hand or digit(s) are taken when the patient first presents in A&E. While a lateral view of the hand will provide a true lateral view of the middle metacarpal, different degrees of rotation of the hand from neutral are required for true lateral views of the remaining metacarpals; 20^o^–30^o^ of supination for the index metacarpal, and approximately 30^o^ of pronation for the ring and small fingers (Shaftel & Capo 2014). If plain radiographs appear normal for a suspected fracture, CT or MRI scans can help diagnose subtle fractures or better delineate intra-articular fractures (Chung 2015).

#### Prophylactic antibiotics and tetanus vaccination for open fractures

Prophylactic intravenous antibiotics are administered immediately in the A&E department for any patient with an open fracture in accordance with local trust antibiotic policy and guidelines (NICE 2017). This is due to the high risk of contamination of the deep tissues. Sloan et al (1987) carried out a prospective trial on 85 patients and found that the infection rate was less than 3% in patients who received antibiotic therapy compared to 30% in the group without antibiotics. More recently, Ng et al (2014) showed that the administration of intravenous antibiotics was the most significant factor in preventing infection in open fractures. In addition, a patient with an open wound and incomplete or uncertain vaccination history should be offered a tetanus vaccine (Ng et al 2014, Unnikrishnan & Bhalaik 2014).

#### Washout and debridement

Washout of the wound is based on the principle of reducing the density of possible microbial contaminants (Kavolus et al 2020). In addition to antibiotic treatment, gross contamination should be removed from the open fracture wound in A&E, under local anaesthetic (Basat et al 2017). A photograph of the wound can be taken for the patient’s record to review wound healing, with patient consent. Following washout, a saline-soaked dressing with an occlusive layer is applied prior to formal debridement (NICE 2017). Wound lavage at this stage can effectively minimise contamination, which can otherwise cause deep tissue infection. Washout should be undertaken, even if this causes a significant delay in surgical treatment (Crowley et al 2007).

#### Venous thromboembolism prophylaxis

Upon admission to the hospital, all patients should be assessed for the need for venous thromboembolism (VTE) prophylaxis (BSSH 2019). VTE is rare in a patient who presents with an isolated hand injury as they are usually still mobile, but the multiple trauma patient with associated hand injuries may have increased risks. VTE assessment will help identify patients at low or high risk of a VTE event (NICE 2022). The British Society for Surgery of the Hand (BSSH) recommends VTE prophylaxis for general anaesthesia (GA) and surgery above 90 minutes. Low-risk patients do not require VTE prophylaxis. Mechanical prophylaxis, such as compression stockings, is recommended for patients with moderate or high risk. Pharmacological prophylaxis, such as low molecular weight heparin, is recommended in high-risk surgery (BSSH 2019).

#### Treatment options

Hand dominance is an indication of how debilitating the injury will be, but other factors including the nature of the injury, occupation and medical comorbidities are all taken into account, to decide the optimal treatment plan for each patient.

Most hand fractures can be treated conservatively with closed reduction, splinting and early mobilisation (BSSH 2021a, Court-Brown et al 2015, Meals & Meals 2013). Closed reduction can take place in the A&E department under local anaesthesia or in the operating theatre under general or local anaesthesia, depending on the severity of the injury and patient comfort. The anatomy of the hand should be taken into account when splinting hand fractures. The interphalangeal joints should be fully extended, while the metacarpophalangeal joints should be positioned in 70^o^–90^o^ of flexion (Dobson et al 2011). This is called the position of safe immobilisation (POSI).

Some basic principles regarding splinting include:

1. The joint above and below the fracture should be immobilised.
2. Splinting should rarely exceed three to four weeks to avoid joint stiffness.
3. Splints should be placed on the side of deformity to prevent redisplacement.
4. Elevate the hand above the level of the heart to reduce swelling (Richards et al 2018).

## Intraoperative management of hand fractures

### Indications for surgery

Open or unstable fractures generally require surgical management (BSSH 2021b, Cheah & Yao 2016, Moore & Varacallo 2021). Other indications for surgery include significant displacement, digital rotation or metacarpal shortening, fractures involving multiple digits or injury to the soft-tissue structures that require surgical reconstruction (Haughton et al 2012, Moore & Varacallo 2021). Surgery should also be considered when the aesthetic appearance of the hand is altered (Haughton et al 2012).

Operative management of closed fractures should take place within seven days following the injury or three days following a failed conservative approach (BSSH 2021a). In open fractures, surgery should ideally be performed within 24 hours of the injury by a hand surgeon (BSSH 2021b).

### WHO Surgical Safety Checklist

The World Health Organization (WHO 2009) Surgical Safety Checklist ensures that vital points prior to the induction of anaesthesia, skin incision and before the patient leaves the theatre are checked. Its implementation has resulted in the reduction of patient deaths and surgical complications across the UK (NHS England 2019). Patient details, such as confirmation of consent, preoperative markings and potential surgical complications, need to be confirmed in the sign in and timeout stages (WHO 2009).

### Anaesthesia

According to NICE, regional blocks are successful in 99% of patients undergoing upper limb or hand surgery, but if unsuccessful, GA is required to proceed with the surgery (NICE 2009, Ode et al 2017). Following anaesthesia, the patient is positioned supine with the affected limb on an arm table, with an angle of abduction <90^o^, to avoid injury to the brachial plexus (Armstrong & Moore 2021, Parekh & Gupta 2021). A brachial plexus block may be used for more proximal upper limb procedures, while local radial, median and ulnar nerve blocks may be used in forearm and hand operations (RCoA 2015, Shanthanna & Rajarathinam 2018). Digital nerve blocks are commonly chosen in finger fractures, due to the absence of systemic complications of a wider anaesthetic block (Napier et al 2021).

### Additional equipment

A tourniquet is applied to reduce blood loss during surgery and keep the surgical field clear (Gallagher et al 2020). The upper limb is exsanguinated distally to proximally, and the tourniquet is inflated up to 250 mmHg for a maximum of two hours (BSSH 2021, Gallagher et al 2020). Prolonged use of a tourniquet and/or excessive pressure may result in peripheral nerve injury and tissue necrosis (Sharma & Salhotra 2012). In cases of digital injuries that require surgical repair, a digital tourniquet is used instead (Kim et al 2020). An image intensifier with Picture Archiving and Communication System (PACS), in which intraoperative images can be stored, should be available (BSSH 2021a).

### Fracture fixation

**Figure 3:**
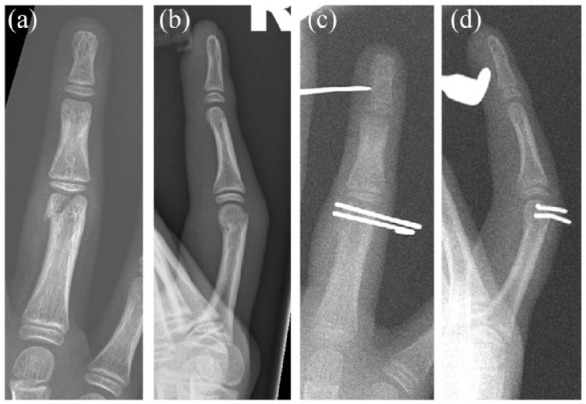
Preoperative AP (a) and lateral (b) views of a proximal phalangeal fracture of the ring finger managed with Kirshner wire stabilisation. Intraoperative AP (c) and lateral views (d) are also shown

**Figure 4:**
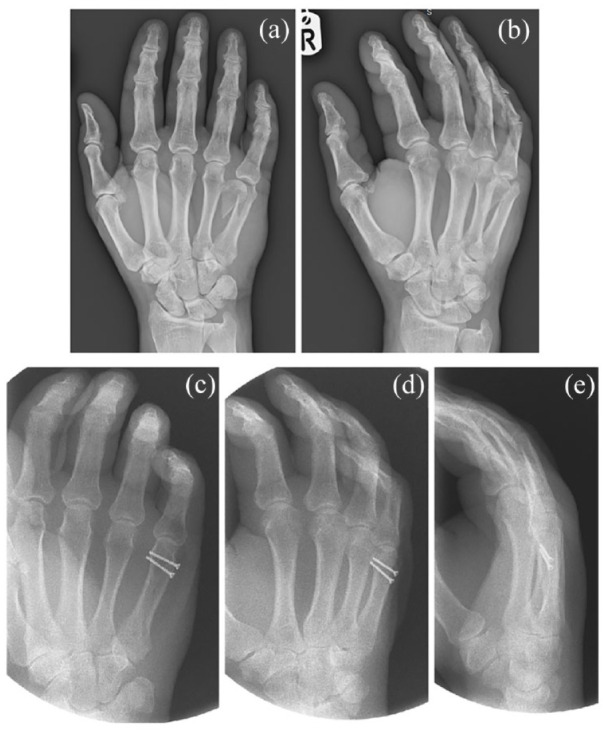
AP (a) and oblique (b) views of a fifth metacarpal fracture managed with screw fixation. Postoperative imaging with screws is shown including AP (c), oblique (d) and lateral (e) views

**Figure 5:**
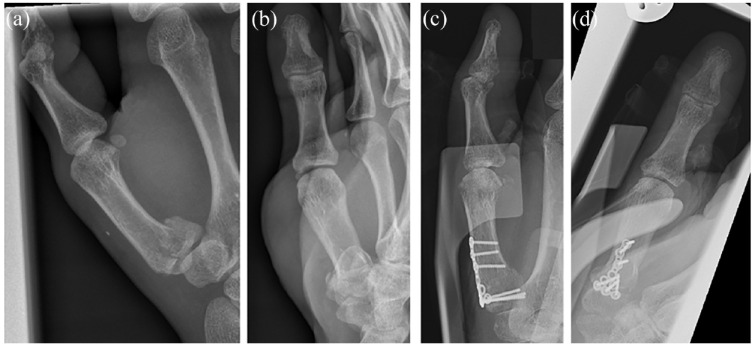
Preoperative AP (a) and lateral (b) views of proximal interphalangeal fracture of thumb managed with screws, plating and splinting. Postoperative AP (c) and lateral (d) views are also shown

Fracture fixation can be achieved through Kirschner wires (K-wires) (see Figure 3), screws (see Figure 4), plates (see Figure 5) or external fixators (Cheah & Yao 2016, Haughton et al 2012, Moore & Varacallo 2021). K-wires do not compress the fracture site, but they are commonly used for preliminary fixation before plating, while they frequently constitute the definitive management of metacarpal and phalangeal fractures.

Internal fixation with screws and plates stabilises the fracture site and allows for early mobilisation due to enhanced stability (Cheah & Yao 2016). In a systematic review, Reformat et al (2018) concluded that the use of K-wires is associated with shorter intraoperative time and longer postoperative immobilisation compared to rigid fixation. However, no difference was found in the active range of motion in both metacarpal and phalangeal fractures, when comparing K-wires to rigid fixation.

External fixators are preferred for small fracture fragments that cannot be fixed well with the aforementioned methods (Cheah & Yao 2016). External fixators are also used to stabilise fractures temporarily while allowing soft-tissue swelling to subside before definitive fixation.

## Postoperative management of hand fractures

A management plan is compiled by the clinical team, which includes the recovery practitioner, an anaesthetist and a surgeon (WHO 2009).

### Immediate postoperative assessment

Following the patient’s surgery, a series of postoperative assessments need to take place in the post-anaesthetic care unit (PACU) and repeated on the surgical ward. First, an ABCDE assessment is carried out in PACU. After initial assessment of airway and breathing, a neurovascular assessment is carried out where possible, within the circulatory assessment. This involves assessing the capillary refill time (which should be ⩽two seconds) on the digit of the operated hand, and the integrity of the peripheral nerves (Figure 2) through sensory and motor function tests. The neurovascular assessment should be documented postoperatively, and the medical team should be contacted if there is deterioration or deviation from the baseline.

If the patient has undergone local anaesthesia (LA), then the sensory assessment should take place after the LA has worn off. At this point, the patient is moved to the surgical ward where the patient’s pain levels should be evaluated, with analgesia prescribed in accordance with local protocols. Oral analgesia is the mainstay of pain control once the patient leaves the hospital. Analgesia prescribed should allow the patient to perform normal activities of daily living, produce minimal side effects and be easy to manage by the patient. An analgesia regime typically involves a combination of oral medications including paracetamol, nonsteroidal anti-inflammatory drugs (NSAIDs) and/or opioid-containing oral analgesics, but this is dependent on both the procedure performed and the patient’s medical history. Regardless of the choice of analgesia, the patient is counselled on what to expect, how to manage and how to use the medications prescribed.

Moreover, an assessment of the patient’s wound is essential to make sure that it remains clean and intact (Wounds UK 2012). When changing the surgical dressings, an aseptic non-touch technique should be used, and the area should be cleaned with saline. Depending on the nature of the injury, the choice of dressings and/or splints should take into consideration the schedule for dressing changes and the early recognition of complications, such as bleeding and infection (Milne 2016). Dressings used can range from basic wound contact dressings (eg: gauze which may or may not be impregnated with paraffin), advanced dressings (eg: hydrocolloid dressings), antimicrobial dressings (eg: silver-containing dressings) or negative-pressure dressings. A Cochrane review (Dumville et al 2014) found no significant difference between one dressing type or another in the prevention of surgical site infection, and the exact dressing choice is therefore guided by surgeon choice.

### Postoperative rehabilitation

Following the operation, the surgeon refers the patient to the hand therapist, who manages the postoperative needs, concerns and expectations of the patient for their recovery. This can be either a physiotherapist or an occupational therapist. Their aim is to direct rehabilitation in a way that best serves the individual patient to meet their expectations and restore optimum hand function (BAHT 2017). The hand therapist also provides emotional and psychological support to the patient and remains in constant communication with the primary health care provider and surgeon (BAHT 2017). Hand therapy should be started as soon as possible and the first appointment with the hand therapist usually takes place five to seven days postoperatively. Overall, the aim is to restore functional capacity within eight to 12 weeks from the time of injury.

Postoperative management of hand fractures aims to balance two contradicting objectives: maintaining stability to allow for fracture healing and encouraging movement so as to permit restoration of function. Immobilisation of the joints around the operated bone will aid healing and reduce the chance of malunion while also providing greater pain relief (Stewart 2019). However, early mobilisation, through physical therapy, reduces the risk of postoperative joint stiffness while increasing the likelihood of better functional outcomes (Luschki 2016).

### The process of hand therapy

Hand therapy is a crucial element of postoperative rehabilitation. It is a specialised process that can be performed by either a physiotherapist or an occupational therapist, and aims to optimise the patient’s hand function (BAHT 2017). Hand therapy comes in four stages: protective, restorative, strengthening and functional (Hays & Rozental 2013). During the protective stages, the aim is to maintain the stability of the affected joints aiming to control pain, minimise swelling and permit proper healing. Minimising swelling is one of the most important aspects of protective management. This can be achieved through hand elevation, buddy strapping and compression techniques and therapeutic massage for lymphatic drainage. A range of motion exercises reduces stiffening (Dorf et al 2010). This process is facilitated by the application of a broad-arm or high-arm sling, with or without a splint, and by the administration of analgesia.

In the first few weeks, the patient is offered a range of exercises, starting from mostly passive, then active-assisted and finally active movements (Hays & Rozental 2013). As the patient’s condition improves and the swelling subsides, there needs to be a constant revision of the splint used, so as to maintain appropriate fit and permit a greater range of movement during therapy. The exercises will progressively increase in difficulty and strength required with the aim of restoring an optimal level of function.

### Adjuncts to postoperative management

There are several interventions that a hand therapist can use to adjust the physiological processes of inflammation and healing, so as to control pain management and accelerate recovery. The application of heat or thermotherapy has been documented as a supportive intervention. Superficial thermotherapy of 41–45^o^C during the protective phase of rehabilitation can increase the blood flow to the affected area, reduce swelling and minimise the concentration of pain mediators (Hartzell et al 2012). In addition, there is also evidence to support the use of ultrasound therapy in postoperative rehabilitation while preventing hypertrophic scar formation (Bashardoust Tajali et al 2012). Furthermore, there is strong evidence to support the role of cryotherapy in managing healing and inflammation (Hartzell et al 2012), as well as the role of electric current application in improving associated neuromuscular function and pain control (Johnson & Martinson 2007). Finally, it is important to note that each patient will be unique, depending on the nature of the fracture as well as the patient’s individual profile, so accessory therapeutic modalities such as the ones mentioned, should be considered to optimise care.

## Conclusion

Appropriate management of hand fractures involves the provision of coordinated care across the preoperative, intraoperative and postoperative settings. Preoperative management relies on an accurate evaluation of the injury. Closed fractures can often be treated with closed reduction followed by splinting and early protected mobilisation. When surgery is indicated, internal fixation using K-wires, screws or plates is generally performed. Postoperative care should involve the input of a specialist hand therapist to maximise the restoration of function.
